# Considerations regarding Interpretation of Positive SARS-CoV-2 Molecular Results with Late Cycle Threshold Values

**DOI:** 10.1128/jcm.00501-22

**Published:** 2022-06-06

**Authors:** Stephanie L. Mitchell, Michael J. Loeffelholz

**Affiliations:** a Medical Affairs, Cepheid, Sunnyvale, California, USA; b Scientific Affairs, Cepheid, Sunnyvale, California, USA; Vanderbilt University Medical Center

**Keywords:** COVID-19, cycle threshold value, RT-PCR, SARS-CoV-2

## Abstract

COVID-19 disease lies on a spectrum, ranging from completely asymptomatic to mild disease to severe and critical disease. Studies have shown that prolonged shedding or sporadic detection of SARS-CoV-2 RNA can occur long after symptom resolution. Adding to these clinical complexities is the demand for testing for SARS-CoV-2 at all stages of diseases, frequently driven by screening of asymptomatic persons, something that traditionally has not been performed for other viral respiratory diseases. This can lead to positive results from nucleic acid amplification tests (NAATs), such as RT-PCR, with late cycle threshold (CT) values near the test’s limit of detection. In this commentary, we review unique attributes of COVID-19 and causes of NAAT late CT values. We provide interpretation considerations as well as strategies to aid in test interpretation.

## HYPOTHETICAL CASE

An asymptomatic adult male presents to an urgent care clinic to be tested for SARS-CoV-2 due to being notified of an exposure to a friend 10 days prior. At the time of presentation to the urgent care clinic, the patient reported a negative rapid antigen test performed at home the day prior. The patient reports no respiratory symptoms in prior 4 weeks. A nasal swab is collected, placed in viral transport medium, and sent to a laboratory to be tested by a reverse transcription polymerase chain reaction (RT-PCR) method. Two days later, results return as positive for SARS-CoV-2 RNA. The clinician questioned the positive result (in an asymptomatic person) and asked the laboratory for additional information on the test result. The RT-PCR test produced a cycle threshold (CT) value of 41 for SARS-CoV-2 gene target “A,” and a CT of 40 for gene target “B.” The test has a cutoff of 42 cycles for each target. Based on this information, the clinician asked the patient to return to the clinic for collection of a second swab for repeat testing by the same RT-PCR method. The patient remained asymptomatic. The second swab, collected 2 days after the first swab, was negative for SARS-CoV-2 RNA. Given the discrepant results, the laboratory decided to retest both specimens. The first specimen tested positive again, with “A” gene target not detected and “B” gene target CT value of 42. The second specimen again produced a negative result. How should the laboratory and the clinician interpret these results?

## OVERVIEW OF SARS-CoV-2 DISEASE

**FIG 1:**
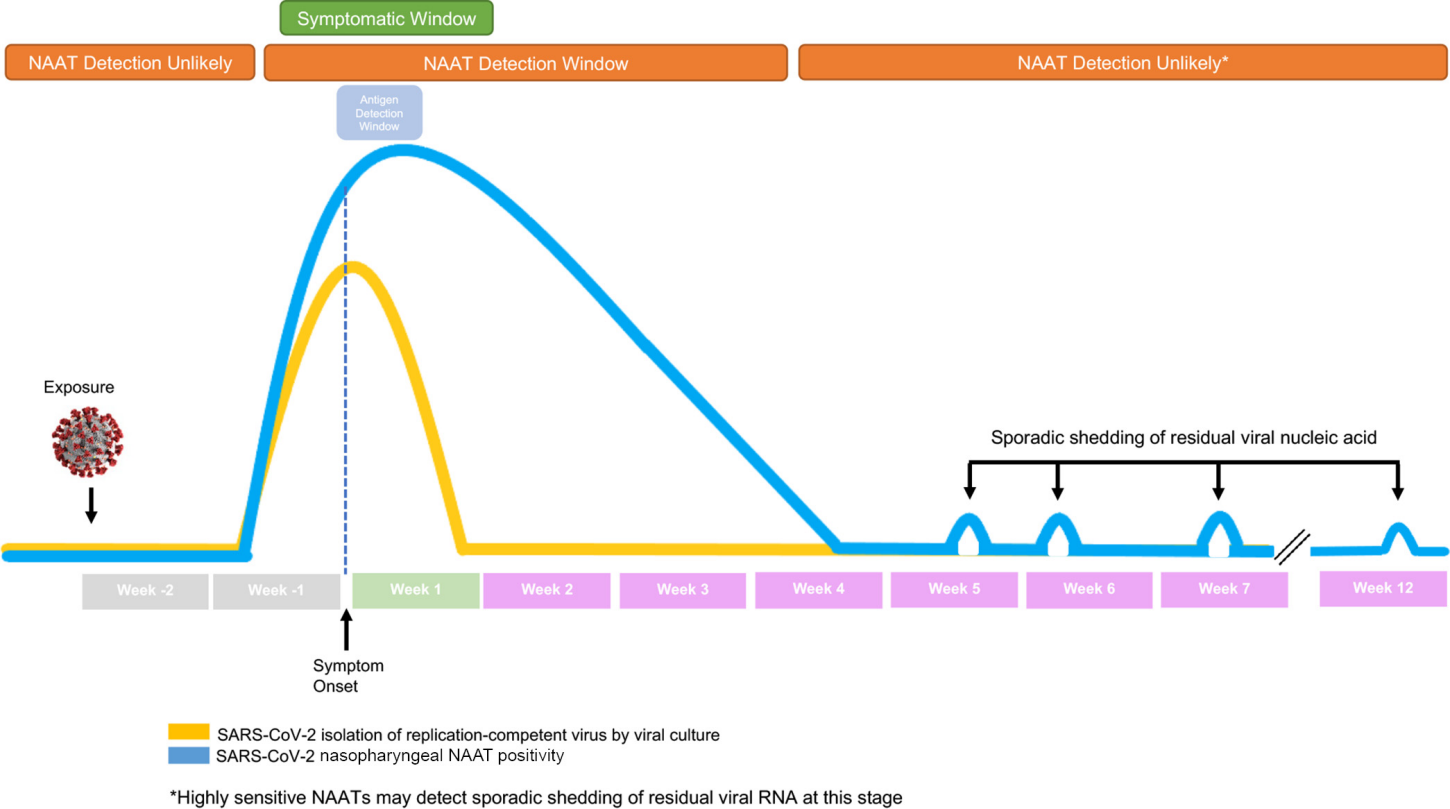
SARC-CoV-2 viral load kinetics and nucleic acid detection.

SARS-CoV-2 diagnostic testing needs have evolved over the course of the COVID-19 pandemic. COVID-19 diagnostic testing needs have evolved over the course of the SARS-CoV-2 pandemic. These changes, along with unique complexities COVID-19 disease, have resulted in new challenges in test interpretation. COVID-19 disease lies on a spectrum, ranging from completely asymptomatic to mild disease to severe and critical disease, which often requires hospitalization. There has also been proposed later disease states, such as COVID-19 related multisystem inflammatory syndrome, but these topics will not be covered here (1). Introduction of vaccines protective against SARS-CoV-2 infection has dramatically shifted the disease spectrum toward more mild disease with less cases of severe and critical disease in the vaccinated population. While disease severity has become milder, the steps of infection kinetics have mostly remained consistent. Stages of SARS-CoV-2 infection and probability of detection can be visualized, in a graphical representation of an asymmetric bell curve (Fig. 1) (2). As the virus begins to replicate postexposure, there is a correlative increase in detectable SARS-CoV-2 nucleic acid; however, this typically begins prior to the development of symptoms. This stage of infected but not symptomatic is commonly referred to as the “presymptomatic” stage of infection and has been shown to play an important role in SARS-CoV-2 transmission (3, 4). The recognition of this presymptomatic stage has likely resulted in recommendations to test individuals with known exposures despite lack of symptoms. While testing during this time may help identify infected individuals early, it is important to note that if tested too early, when viral nucleic acid concentrations are very low, even the most sensitive test may not detect SARS-CoV-2.

Around the peak of viral replication, symptoms often emerge. This symptomatic stage is the most common time when patients present to care and thus are tested. It is also the most likely time when viral nucleic acid can be detected by most diagnostic tests. However, after this peak, viral loads of replication-competent virus (e.g., infectious virus) drop quickly, with each day postsymptom onset reducing the likelihood of nucleic acid detection and a positive test result. This window ranges from around 5 to 7 days. Once outside the approximate 1-week window postsymptom onset, viral loads of active replicating virus are near nondetectable. This also typically correlates with disease resolution in otherwise healthy patient populations. One somewhat unique state of COVID-19 is the seemingly high number of asymptomatic infections, which has been reported to be around 20% to 30% of cases, in both the pre- and postvaccination era, although data postvaccination is scarce (5–7). This is in stark contrast to some other respiratory viruses, which are thought to cause mostly symptomatic infections; however, this assumption may be due to lack of testing in an asymptomatic population for other respiratory viruses (8). As such, this represents a diagnostic challenge as those that may be infected but without noticeable symptoms will not present to care or be tested. While the significance of SARS-CoV-2 transmission from asymptomatic individuals has been shown to occur in these patients without disease and thus, may go unidentified by exposed individuals and contract tracing efforts (9). Therefore, this silent transmission by an asymptomatic patient results in unclear and confounding interpretation of a positive diagnostic test in an asymptomatic individual with no identified known exposures.

Due to these complexities, this has resulted in significant demands for testing for SARS-CoV-2 ranging from symptomatic diagnostic testing to asymptomatic screening and surveillance all along the infection curve, something that traditionally has not been performed for other viral respiratory diseases. Notably is the demand for SARS-CoV-2 screening in various contexts. For those individuals that previously had COVID-19, this often means screening tests are performed at an unknown stage of new disease, if infected, and many weeks to months postdisease resolution, falling on the far right of the infectious peak in the infection curve graphic (Fig. 1). For most respiratory viruses, it was generally accepted that viral nucleic acid clearance occurs relatively rapidly. However, with more wide-spread use of highly analytically sensitive nucleic acid amplification tests (NAATs) for detection of respiratory viruses, especially for SARS-CoV-2, this has become a topic of debate. As shown in Fig. 1, test positivity kinetics by NAAT is not identical to viral load as determined by viral culture and can remain positive outside the 5- to 7-day viral culture positive windows (10–13). For SARS-CoV-2, despite disease resolution after around 1-week postsymptom onset, studies have shown that prolonged shedding or sporadic circulation of viral RNA can occur for many months postsymptom resolution and these results often have late CT values (Fig. 1) (14, 15). This begs the question of whether this detection represents replicating virus with potential of transmission or residual noninfectious fragmented viral RNA being released as a result of immune clearance (12, 16, 17). Indeed, all molecular tests have the inherent limitation of only detecting the presence or absence of the targeted nucleic acid. Therefore, NAAT detection does not distinguish between nucleic acid detected of live, dead, or cell-free nucleic acid present in the sample and thus can confound test interpretation. These late CT SARS-CoV-2 positive samples from patients with resolved COVID-19 have been shown to not be positive by viral culture, suggesting no active replicating virus is present. In fact, the World Health Organization (WHO) and Centers for Disease Control and Prevention (CDC) have published guidance documents that suggest that patients are not likely to be infectious after 10 days from the start of symptoms for mild cases and up to 20 days for severe disease (18, 19). A consideration for these conclusions is the inherent challenges of adequate viral culture methods, such as proper sample transport and ability to detect cytopathic effect or progeny virus (20). However, an argument can be made that a viral culture may more accurately reflect clinically relevant viral loads that correlate with disease and transmission. Based on these data, the CDC now recommends avoiding repeat NAAT testing in immunocompetent asymptomatic individuals for at least 90 days from the previous positive result (21). Despite this “no NAAT within 90 days” recommendation, testing during this time still occurs. Due to those considerations listed above, obtaining a positive test result during this time may still represent true disease and should not automatically be disregarded as detection of noninfectious residual viral RNA.

## CAUSES OF LATE CT POSITIVES

For the purposes of this commentary, late CTs are defined as a CT near the upper limit of detection for that specific test. Typically, this is around 3 to 5 CT from the CT cutoff threshold and often is in the high 30s to mid-40s (22). Factors that can cause late CT values in SARS-CoV-2 RT-PCR tests include the timing of specimen collection relative to the date of exposure or duration of symptoms, collection of specimens after starting antiviral therapy, specimen quality or collection variability, presence of PCR inhibitors in the specimen, and specimen transport and storage conditions to include freeze/thaw cycles. Testing of pooled specimens (performing one test on a combined pool of specimens from several persons) may also contribute to late CT values (23).

### Timing of specimen collection.

As with other respiratory virus infections, SARS-CoV-2 viral loads decrease following their peak near symptom onset (Fig. 1). Retesting of known positive patients can be associated with later CT values (24). The emphasis on asymptomatic screening as a means to control the COVID-19 pandemic has resulted in many persons being tested fortuitously during the presymptomatic and more likely, postsymptomatic phase when SARS-CoV-2 load can be very low (late CT values) (25, 26). As described above, prolonged shedding of SARS-CoV-2 RNA frequently occurs following infection. This shedding can be at very low loads and sporadic (15).

### Prior antiviral therapy.

Biancofiore et al. reported a significant decline in SARS-CoV-2 viral load, and in some specimens with late CT values, 7 to 14 days after remdesivir treatment (27). Anti-SARS-CoV-2 monoclonal antibody therapy can also significantly reduce viral load (28).

### Specimen type.

SARS-CoV-2 viral loads have been reported to vary by specimen type, but this may be further complicated by SARS-CoV-2 variant. For example, the Omicron variant appears to have high viral loads in saliva compared with earlier variants (29). Despite this, nasopharyngeal (NP) specimens are still considered the best specimen type for SARS-CoV-2,and is the sample to which all other samples are compared (30).

### Presence of PCR inhibitors.

Potential PCR inhibitors in clinical specimens include blood, mucous, and exogenous substances such as pharmaceuticals (31). The inhibitory effect can be partial, resulting in delayed CT values.

### Specimen quality.

Poor-quality nasopharyngeal swab specimens (as measured by amplification of a human endogenous reference gene) are associated with later SARS-CoV-2 CT values (32). The challenge of specimen quality can be compounded by inconsistent specimen collection guidance (33).

### Specimen stability/transport.

The stability of SARS-CoV-2 RNA in specimens can vary by transport medium and environmental conditions (34, 35). Multiple freeze/thaw cycles of specimens may decrease the sensitivity of SARS-CoV-2 RT-PCR tests (36). These conditions can lead to target degradation during the preanalytical phase, as well as late CT values. Users should establish specimen stability or follow manufacturers’ instructions for specimen collection, handling, and storage.

## INTERPRETATION CONSIDERATIONS

### Symptomology.

In the presence of symptoms consistent with COVID-19, any positive RT-PCR result regardless of CT value should be considered diagnostic. Both symptomatic and asymptomatic infections can have a wide range of viral loads (15). Given the multiple variables independent of symptoms (e.g., specimen quality, specimen source, PCR inhibitors) laboratorians and clinicians should avoid inferring too much from a single positive CT value. While severe COVID-19 cases generally have higher viral loads (earlier RT-PCR CT values) than mild cases, there is considerable overlap among individual results (4, 37). Additionally, noninfectious etiologies or underlying respiratory disease may cause similar symptoms as COVID-19, which may confound the clinical assessment of symptomology.

### High-risk individuals.

Immunocompromised patients may be more likely to have persistent shedding (either residual RNA or infectious virus) than immunocompetent persons, with a longer time to PCR clearance, presumably due to suppressed immune function, such as reduction in B-cell function (38). While the data do not suggest that immunosuppressed patients are more likely to have persistently lower viral loads in general, the longer time for PCR clearance and prolonged shedding suggests greater random chance to obtain collect specimens with late CT values.

### Exposure history and vaccination status.

Breakthrough SARS-CoV2 infections following vaccination occur but tend to have lower viral loads than those in unvaccinated persons (39). However, the impact of vaccination on breakthrough infection viral load may be variant dependent. A study of breakthrough infections caused by the Delta variant of SARS-CoV-2 showed similar peak viral loads between vaccinated and unvaccinated subjects (40). Specimens collected from vaccinated individuals with breakthrough infections can have late CT values, with both Delta and Omicron variants (41–43).

### Symptom duration and disease state at presentation.

As previously discussed, the peak SARS-CoV-2 viral load roughly corresponds with symptom onset, and then gradually declines to undetectable antigen within a few days and undetectable RNA by highly sensitive nucleic acid amplification tests within 7 to 14 days in most persons. RT-PCR is often positive in the presymptom period and occasionally well beyond 2 weeks after symptom onset in those who exhibit long-term shedding. With this in mind, it is important to assess the disease state at presentation, and if symptoms are present, to know their duration. Specimens collected more than approximately 2 weeks after symptom onset are likely to have late CT values (44).

### RT-PCR CT correlation with culturable virus and molecular markers of active infection.

Given the relatively high rate of asymptomatic COVID-19 and long-term RNA positivity in some persons, an unexpected positive result, such as the case presented in this commentary, can raise questions around the epidemiological significance or contagiousness. Several investigators have examined the correlation between PCR CT values and the ability to isolate virus in cell culture. Singanayagam et al. were able to recover virus from only 8% of specimens with CT values >35 (45), while La Scola et al. were unable to isolate virus from any specimens with CT values ≥34 (10). Reporting a different CT breakpoint, Bullard et al. were unable to isolate virus from specimens with Ct values >24 (46). Others have used molecular markers of infectivity, such as SARS-CoV-2 subgenomic RNA (11) or minus-strand RNA (47) as surrogates for actively replicating SARS-CoV-2. Hogan et al. showed significant correlation of minus-stand RNA positivity with standard RT-PCR CT values (47) suggesting that there is less replicating virus at late CT values.

### Disease prevalence and pretest probability.

As with all in vitro diagnostics (IVDs), the performance of a test depends on not only on the analytical sensitivity and specificity but also the prevalence of disease at the time the test is performed. Disease prevalence is not constant and may change seasonally, as with many respiratory viruses, but also may differ among patient populations or over time. Importantly, testing when disease prevalence is low increases the risk of a positive test results being a false-positive (48).

Prevalence can sometimes be referred to pretest probability. However, pretest probability may also take into account patient specific factors, such as symptomology, past medical disease, and exposure history. Using the Bayes’ theorem, pretest probability, in context with disease prevalence and analytical test performance characteristics, can allow for estimation of the probability the patient has the disease before a test is even ordered (49). Knowing this probability is fundamentally important for proper test interpretation and may also guide clinicians as to whether to even perform the test or not. Test results are placed into clinical and epidemiological context, which may be considered one of the most important factors in diagnostic test interpretation. As such, disease prevalence and pretest probability are important influencers in the clinical performance of the test.

### Test performance and intended use.

SARS-CoV-2 NAATs include isothermal amplification methods such as transcription-mediated amplification (TMA), loop-mediated isothermal amplification (LAMP), helicase-dependent amplification (HDA), Nicking Endonuclease Amplification Reaction, and others. Other NAATs utilize RT-PCR, including real-time and nested PCR. Only real-time RT-PCR will produce CT values. At the time of this writing, there are no EUA authorized SARS-CoV-2 NAATs with a quantitative claim in their intended use. Therefore, the reporting and use of CT values to make patient management decisions may be considered off-label. CT values produced by qualitative RT-PCR tests may vary significantly between and even within methods (50).

The claimed limit of detection (LOD) of a RT-PCR test is important to consider in the context of late CT values and their interpretation, including repeat testing. The LOD is defined as the lowest amount of analyte in a sample that is detected with acceptable certainty (usually at least 95% of replicates tested). For diagnostics, LOD is critical as this provides useful information regarding analytical reproducibility and reliability, as those concentrations above the LOD are generally highly reproducible. However, imprecision in results and stochastic performance is often seen at near or sub-LOD concentrations (51). This imprecision is due many factors, including increases in background noise and the reduction in the probability of detection, as predicted by binomial test models. The farther the concentration goes past the LOD, the less likely a test will be able to reproduce analyte detection. As a result, tests are inaccurate at sub-LOD levels, whereby a high number of repeat tests are needed to detect an analyte. If late SARS-CoV-2 CT values represent sub-LOD concentrations of RNA target for a particular method, repeat testing of a specimen will produce a certain level of nonreproducibility.

### Clinical versus analytical specificity.

In the setting of late CT positives for SARS-CoV-2, the distinction between analytical specificity and clinical specificity is worth highlighting. Analytical specificity is an assessment of how well a test detects only the desired analyte while avoiding detection of undesired analytes. Clinical specificity, on the other hand, is how well a test can correctly identify individuals without disease. The detection of the SARS-CoV-2 target at late CTs is sometimes considered to be a “false-positive test result.” However, knowing there are two definitions of specificity, it is prudent to consider what is meant by “false-positive.” Analytically speaking, the likelihood of an analytical false-positive (e.g., detection of a nonspecific analyte) is very low given the numerous analytical studies performed during test development to ensure analytical specificity is acceptable. In these late CT cases, therefore, it is likely that the concern is for a clinical false-positive (e.g., detection of the analyte is correct but did not correctly identify disease). For SARS-CoV-2 this represents a “gray area” for diagnosis given the complexities in disease characteristics/kinetics along with dramatic fluctuations in prevalence and pretest probabilities, as mentioned above.

## STRATEGIES FOR LATE CT POSITIVES

Given the muddy waters in which we find ourselves when interpreting a late CT positive SARS-CoV-2 NAAT test results in asymptomatic patients, strategies have evolved to help provide clarity and aid in test interpretation and downstream clinical management.

One of the more widely recommended strategies is to collect a second sample and test using the same or analytically equivalent NAAT SARS-CoV-2 test as the first test (52, 53). Based on infection kinetics, most guidelines recommend waiting at least 1 to 2 days before obtaining the second sample. This 1- to 2-day wait is advantageous for a few reasons. This allows time for gathering of additional clinical context, such as reassessment/development of symptoms and exposure history. Additionally, testing of a second sample avoids any analytical test limitations when samples fall at or below the LOD, as described above. If active replication is occurring, a 1- to 2-day wait allows more time for viral loads to increase versus no change if no viral replication is occurring. Therefore, a second positive test result with potentially a reduction in CT value would indicate true infection. However, if the second test is either positive but with a similar late CT value or is negative, in the absence of clinical factors that would otherwise raise clinical suspicion of true infection, this set of test results can be regarded as residual RNA detection (53). This approach is not without limitations, as sample bias (as previously described), adequacy, or differences in testing platforms may play a role in discordant results between two samples. Therefore, it is imperative that sample collection, handling, and transport integrity remains intact for reliable test interpretation and testing ideally be performed using the same platform. In some cases, testing for presence of antibody to SARS-CoV-2 nucleocapsid may be considered to aid in NAAT interpretation, which may indicate previous exposure to a native SARS-CoV-2 infection. However, antibody testing for such purposes is rarely done, especially outside the hospital setting. Taken together, testing a second sample may aid in the interpretation of initial late CT positive results and differentiate individuals with remote infection who are likely to be noninfectious from those with acute infection, at the time of testing.

Other strategies to address this issue have also been used; however, these come with considerable limitations and are generally not recommended. One option many labs may employ is simply repeat testing using the same sample. However, as noted above, these late CT values are often near or beyond the test’s LOD, where inherently, the test has significant reduction in the reproducibility of analyte detection. Therefore, when testing samples that have sub-LOD concentrations, a single repeat test may not be mathematically sufficient to reproduce the initial result. Obtaining a repeat negative test result on the sample with sub-LOD viral RNA does not confirm that the initial positive results was analytically false.

More alternative strategies include modification of the test parameters, such as changing the threshold cutoff values or alternating test interpretation based on CT values. This is based on some publications that state that NAAT with CT > 34 do not have detected virus by viral culture and thus corresponds to patients that are not likely to be infectious (16, 17, 54, 55). However, it is important to remember that CT values cannot be compared across platforms, as the absolute number is influenced by test methodology, design, cycling parameters, and data analysis criteria. Therefore, the use of a generic “CT > 34” for any test may not be appropriate. There have also been proposals of different interpretations using CT values, which depending on the test results, trigger additional actions (56). Yet another option may be to report the CT value along with the qualitative test result into the patient chart to allow interpretation of the CT value by the clinician. While these may seem like viable approaches, it is important to consider that such modifications are off-label for many SARS-CoV-2 diagnostic tests claims and intended use, which to date are authorized for only the qualitative detection of the virus, and therefore, are not recommended. If desired, the responsibility and risk of determining an alternative test interpretation or cutoff for that specific test and establishing acceptable analytical and clinical test performance falls to the performing laboratory. Another consideration is that due to disruption in supply chain, many laboratories have implemented numerous IVD or laboratory developed testing platforms to meet testing demand. It is important to note that not all NAAT SARS-CoV-2 diagnostics provide a CT value. In multiple testing platforms for SARS-CoV-2 diagnostics, an in-depth understanding of how such modifications or reporting of CT values may impact laboratory, information technology, and clinician workflow is critical. Additional publications regarding the use of CT values for qualitative SARS-CoV-2 tests have provided guidance around this topic, and generally recommend against such modifications (50, 57–59).

## CONCLUSIONS

How should the laboratory and the clinician interpret the test results in the case described? When taken into context with the considerations described in this text, the test results in this case are likely consistent with the presence of SARS-CoV-2 RNA at a concentration near or below the limit of detection of the RT-PCR test. At the CT values reported by the RT-PCR test, it is not unexpected that the antigen test was negative (60). The RT-PCR results from the two swabs do not indicate an ascending viral load, which would be observed for a patient in the presymptomatic period and suggest that the patient is unlikely to be contagious. An asymptomatic infection acquired from the exposure 10 days prior could explain the results. Alternatively, an undisclosed infection 2 to 3 months prior could also explain the results. Collection of additional history, including vaccination status, travel, ill-household members, and local prevalence of disease would be helpful information for proper test interpretation.
